# Perceptions about *pasung* (physical restraint and confinement) of schizophrenia patients: a qualitative study among family members and other key stakeholders in Bogor Regency, West Java Province, Indonesia 2017

**DOI:** 10.1186/s13033-018-0216-0

**Published:** 2018-06-25

**Authors:** Nenden Hikmah Laila, Renti Mahkota, Tri Krianto, Siddharudha Shivalli

**Affiliations:** 1 Lebak Distric Health Office, Rangkasbitung, Lebak, Banten 42311 Indonesia; 2 Department of Epidemiology, Faculty of Public Health, Universitas Indonesia, Depok, 16424 Indonesia; 3 Department of Health Education and Behavioral Sciences, Faculty of Public Health, Universitas Indonesia, Depok, 16424 Indonesia; 4 Non-Communicable Diseases Regional Technical Advisor, South-East Asia Regional Office (SEARO), TEPHINET, A Program of the Task Force for Global Health, Inc., Decatur, GA 30030 USA; 5 Department of Public Health, Yenepoya Medical College, Yenepoya University, Mangaluru, Karnataka 575018 India

**Keywords:** Schizophrenia, *Pasung*, Qualitative

## Abstract

**Background:**

The UN resolution recommends treating all mentally ill patients with humanity and respect. However, social stigma continues to prevail for patients with schizophrenia. Physical restraint and confinement of the mentally ill is a well-known phenomenon in Indonesia and is termed as *pasung*.

**Objective:**

To explore the perceptions of family members of patients of schizophrenia and other key stakeholders concerning *pasung* in Bogor Regency, West Java Province 2017.

**Methods:**

This qualitative exploratory study was conducted in Bogor Regency, West Java Province from May to June 2017. This study involved 12 key stakeholders including family members, neighbors, community leaders, and mental health officers. In-depth interviews were conducted with family members (n = 3) who practiced *pasung* for patients with schizophrenia and key informant interviews of neighbors, community leaders (two household heads and one from a health cadre) (n = 3) and mental health officers of *puskesmas* (three midwives). Data triangulation was performed by interviewing residents and mental health workers. Content analysis was conducted and themes were identified based on valid inference and interpretation.

**Results:**

Family members and society in general perceived that *pasung* is necessary for security reasons due to the patient’s aggressive behavior such as physical violence to neighbors, stealing food etc. According to community leaders, families often do not respond to patient’s request to be released from *pasung*. Family members had financial constraints to seek mental healthcare and were also dissatisfied with available services. Healthcare providers highlighted the poor knowledge and prevailing misconceptions about schizophrenia in the community.

**Conclusion:**

Concurrent efforts to strengthen basic mental health services and health education regarding schizophrenia, prevalent misconceptions, and importance of timely and appropriate treatment are needed, especially in rural settings.

## Background

According to Article 1 of the Universal Declaration of Human Rights by the United Nations in 1948, all individuals are free and have equal rights and dignity. This protects the fundamental rights of individuals with physical or mental disabilities [1]. Globally, more than 21 million people are affected by schizophrenia. This is a severe mental disorder characterized by distorted thinking, perceptions, emotions, language, sense of self and behavior [2]. Unfortunately, schizophrenia is associated with stigma and leads to discrimination of the affected in the community. This can limit their access to healthcare, education, employment and quality of life [2]. Stigma is a social problem in which the environment negatively labels one’s situation and condition including attitudes of rejection, denial and isolation. Poor knowledge of mental disorders, misconceptions, lack of motivation, poor access and unavailability of mental healthcare services contribute to the problem [3–5].

More than 50% of people with schizophrenia do not receive appropriate mental healthcare [2]. Nearly 90% people with untreated schizophrenia live in low and middle income countries [2]. Unfortunately, the condition of people with mental disorders in Indonesia is unsatisfactory. Poor availability and access to basic mental health services have been highlighted [6]. Family members often take regressive measures on patients with mental disorder due to stigma induced stress and sense of helplessness [3–5]. One of the regressive measures is physical restraint and confinement of the affected person and is commonly referred to as *pasung* in Indonesia [5]. *Pasung* is common in developing countries, including Indonesia [7]. In addition to the use of wood or leg chains to restrict movements, *pasung* also involves confinement and neglect [8]. Suicidal tendencies are common among untreated patients with schizophrenia under *pasung* [9, 10].

According to the Basic Health Research of Indonesia-2013 (*Riset Kesehatan Dasar* known as *Riskesdas*), 14.3% of Indonesian households have a patient with a mental disorder and a majority are in rural areas [11]. The prevalence of mental disorders in West Java Province is 20%, the highest in Indonesia [5]. In West Java, the prevalence of severe mental disorders is 1.6 per mile [11]. Bogor is one of the regencies in West Java Province listing 1323 schizophrenia patients (2016–17) registered in the report of people with mental disorders [12]. Seventy-five (5.6%) of the 1323 schizophrenia patients in Bogor Regency were under *pasung* [12]. Exploring family members’ and other key stakeholders’ (community members and healthcare providers) perceptions on *pasung* would help to understand the ‘emic’ view of the issue.

## Objective

To explore the perceptions of family members of patients of schizophrenia and other key stakeholders concerning *pasung* in Bogor Regency, West Java Province 2017.

## Methods

### Study design and setting

This study used a qualitative exploratory design with semi-structured in-depth and key informant interviews. Bogor is one of the 17 regencies of West Java Province, Indonesia with a total population of 5.13 million. This is subdivided in 40 districts (*Kecamatan*). The public health infrastructure consists of one district hospital and 101 primary health centers (*Pusat Kesehatan Masyarakat, puskesmas*). *Puskesmas* is a part of the three-tier system public healthcare system in Indonesia. In addition to maternal and child health care, they provide preventive, promotive, and curative healthcare services.

### Data collection

This qualitative study involved 12 key stakeholders. In-depth interviews were conducted with family members (n = 3) who practiced *pasung* for patients with schizophrenia and key informant interviews of neighbors, community leaders (two household heads and one from a health cadre) (n = 3) and mental health officers of *puskesmas* (three midwives).

Keeping the study objectives in mind, authors selected participants who could provide information by virtue of experience or knowledge. Hence, purposive sampling was used to select the study participants. Three patients with schizophrenia in *pasung* were selected purposively from the *pasung* register of Bogor Regency Health Office and one of the adult family members was invited to participate. Heads of the families in the neighborhood, community leaders and midwives working in the nearest *Puskesmas* were invited for key informant interviews.

Semi-structured interview guidelines were prepared by reviewing the existing literature [3–6, 8–10] and the following topics were included: nature and duration of *pasung* followed, perception about *pasung*, reasons for *pasung* and perceptions about the available healthcare services. Before the interview of family members, basic details of patients with schizophrenia in *pasung* (age, sex, marital status, nature, and duration of *pasung*) were asked. For family members and neighbors, and community leaders interviews were conducted in their homes. For mental health officers, interviews were conducted at their respective *puskesmas.* Each interview lasted for about 30–40 min. All the interviews were conducted by the first author (NHL) and a note taker was also present during the interview to record the comments in detail.

### Data analysis

Interview transcripts were prepared by the first author on the same day and qualitative content analysis was conducted. Study objectives related to content units were identified and pooled in one unit of analysis. Frequently occurring content units were coded and classified in emerging themes. Participant quotes were selected to illustrate emerging themes and were translated to English.

## Results

Two mothers and one sister of a patient with schizophrenia with *pasung* participated in indepth interviews.

Table 1 shows the details of schizophrenia patients in *pasung*.

**Table 1:** Demographic details of patients of schizophrenia regarding *pasung* in Bogor Regency, Indonesia 2017

Age	Sex	Marital status	Relapse	Nature of *pasung*	Duration of *pasung*
25 years	Male	Widower	Once	Patient was tied up and chained on both legs while undergoing alternative treatment	10 days
34 years	Male	Unmarried	18 times	Patients were tied up and chained in a vacant house on a dirty floor	3 years
16 years	Male	Unmarried	Often	Patients were tied up and chained in goat cages every night	2 months

Based on the content analysis, the following items were identified.

### Perceptions of *pasung*

According to family members, neighbors and community leaders, security/protection of the patient and others was the main reason for *pasung*. Most displayed aggressive behaviors such as physical violence to family members and neighbors. They damaged the furniture, neighbors’ environment/garden, stole food, threw things, broke glass etc. When not in *pasung*, patients wandered out of the house. Under such circumstances, family members felt insecure and helpless. All these culminated in *pasung* of the patient with schizophrenia (Table 2).

**Table 2:** Selected codes and themes that emerged while exploring the perceptions about *pasung* among key stakeholders in Bogor Regency, Indonesia 2017

Selected code	Theme
Violent behavior Destructive behavior Damage to garden/surroundings Only alternate measure It is ok to lock him up	Perceptions about *pasung* Necessary to protect patient and others from his/her own aggressive behaviors An acceptable pragmatic solution
Not available in rural areas Not satisfied No money for transport Poor family Can’t feed the patient	Mental health services Poor access Financial constraints
Reduced aggression is healing *Pasung* is fine	Knowledge of schizophrenia Poor knowledge and prevailing misconceptions

*“Yes if AS didn’t get feds, he gets angry. If late in feeding AS always damaged goods, slammed items, threw things… glasses were broken”. He calms down when he is in pasung* (Mother of AS).

*“When AJ is not in pasung he likes wandering around… likes going into neighbor’s homes, and then stealing some food”* (Community leader).

*“In the past, AJ liked to destroy people’s gardens while carrying a machete”* (neighbor).

Family and community members feel that *pasung* is the only alternative so as to protect others and the patient him/herself from aggressive and destructive behaviors. *Pasung* was perceived as a practical solution for patients with mental illness and was an accepted norm in the community. Sometimes, patients expressed the desire to see a doctor or to be shifted to a mental hospital, but families tended to ignore the request due to various reasons.

*“There is no other alternative except pasung because most of the time nobody is at home, father is being treated in the hospital and mother is also sick”* (Family member).

*“….better if he is in the cage or locked up in the room, it is okay. So he will not harm others”* (Neighbor).

### Financial issues

Families cannot bear the mental healthcare cost. Sometimes, they could not afford the transport cost even when they had health insurance. In addition, families could not meet the feeding expenses of over-eating patients. Because of all these reasons, families were unable to meet even their basic needs.

*“Sometimes the obstacle is the cost, no funding.., if we refer the patient to a hospital, maybe they have health insurance, but usually they object because they don’t have money for transport”* (Health officer).

*“We don’t have much money, AS always asks for meals..in one day it can be 14 times”* (Family member).

*I asked for help from community leaders, from the youth, big family… but nobody cares,*

*I’m so confused… *(Family member)*.*

### Dissatisfaction with mental health services

Access and availability mental health services were dismal in rural areas. A lack of trust was evidenced and often treated patients had a relapse. Mental health services are relatively better and well-coordinated in urban settings consisting of medical care and psychological counseling to change the behavior. Families often sought alternative treatments which were easily available.

*“He was already referred to the hospital twice; apparently after going home he relapsed, and eventually his family asked to be treated by alternate therapy”* (Health officer).

*“AS stole food from a neighbor’s house after being discharged from the hospital” *(Community leader)*.*

*“In fact, within 10* *days AR recovered, recovered completely by alternate treatment. My sadness disappeared because AR was healed”* (Family member).

### Limited knowledge of schizophrenia

The community had limited knowledge of schizophrenia and how to take care of patients with schizophrenia. On the contrary, misconceptions and misunderstanding were common. Stopping aggressive behaviors and obeying parents were considered as signs of healing even when the patient was in *pasung*.

*“The most important for me is healing of AR even if he in pasung, no problem. It is okay, that’s the duty to treat”* (Family member)

*“Yes, leave it alone. He would not be in pasung if he didn’t fall sick ….”* (Community leader)

## Discussion

In the study setting, family members and community leaders perceived *pasung* as a necessary measure due to patients’ aggressive and destructive behaviors. Financial constraints and dissatisfaction with existing mental health services were the reasons for not seeking mental health care. Poor knowledge and misconceptions about schizophrenia were prevalent in the study setting.

Violation of human rights is commonly seen among patients with mental disorders [2, 13]. Lack of economic productivity, loss of meaningful social roles and adult decision-making capacity are the reasons for ill treatment [14]. Schizophrenia is now considered as a mental health problem of global priority [15]. *Pasung* or chaining or shackling or physical restraint is one of such human rights violations. *Pasung* appears to be very common among patients with schizophrenia, although seen among other mental disorders in Indonesia and other countries [16–19]. In this study, patient’s aggressive and destructive behaviors were the main reason for instituting *pasung*. Family members and community leaders perceived it as a pragmatic solution to protect patients, family members, and neighbors. However, they never openly discussed the stigma associated with schizophrenia as a reason for *pasung*. Similar findings of physical restraints were reported from *Samosir* and *Aceh*, Indonesia [16, 17, 20] and other countries [19, 21]. Other reasons for *pasung* or physical restraint were wandering by the patient (in this study) and to access treatment [21]. Physical restraint was not restricted to domestic places but also reported in traditional or spiritual healing centers and mental hospitals [22, 23]. Such social abandonment varies drastically across cultural geography. In countries like Indonesia, India, China, Ethiopia etc. [16, 17, 19–21] people with mental disorders are physically restrained in domestic space whereas in the US they are more likely to be expelled to the street [24].

In this study, financial constraint was the most prominent reason to discontinue treatment. In addition, dissatisfaction with existing healthcare services was prevalent and was attributed to relapse despite treatment. Study participants perceived that mental healthcare services in rural parts of Bogor Regency were not on par with urban mental healthcare facilities and many could not bear the travel costs. Indonesian national health insurance (*Jaminan Kesehatan Nasional*, JKN) administered by the BPJS Social Insurance Administration Organization (*Badan Penyelenggara Jaminan Sosial,* BPJS) covers mental health services to all the Indonesians. However, it does not cover the costs of transport and accommodation of the patients’ entourage. These hinder family members seeking timely treatment and availing benefits of JKN. Similar to the present study, a major concern was travel costs as no nearby mental healthcare facility are available in *Aceh*, Indonesia [16]. Impacted by the uptake of JKN where primary care clinics were conveniently located, access was often complicated by long waiting times and short opening hours. Lower levels of trust with primary care doctors was observed especially compared with hospital and specialist care. Also, a sense of anxiety existed that the current JKN regulation might limit their ability to access hospital services guaranteed in the past [25]. Due to health system related factors, unaffordability and patient’s aggressive behavior, families were left with no choice but to institute *pasung*.

In this study, caregivers attributed overeating by patients of schizophrenia as another reason for economic hardship. Existing literature suggests that 8–12% and 6–16% of patients with schizophrenia have night eating and binge eating disorders, respectively [26–30]. Even this requires early detection and multidisciplinary management [31].

Schizophrenia can be effectively treated by medicines and psychosocial support. Facilitation of assisted living, supported housing, and employment are effective management strategies for schizophrenia [2]. The Global Movement for Mental Health (a network of individuals and organizations to improve services for people living with mental health problems) has highlighted the scaling up of mental health and development of policies and legislation to enhance access to mental health care and protection of human rights [32]. Poor access and availability of quality mental health care force the family members to seek alternative treatments such as traditional or spiritual healers [32].

Unfortunately, in this study, *pasung* was a socially acceptable alternative measure for family members and society. On the contrary, isolation, neglect and lack of treatment further worsen schizophrenia and such patients are likely to have suicidal tendencies [9, 10]. In addition, physical restraint itself can cause serious complications including aspiration, infection and even death [33]. An urgent need exists to build basic mental health services system in the district. Related studies have shown that the practice of physical restraint could be eradicated by scaling up services to provide effective, accessible, and affordable mental healthcare for the needy [19, 34–36]. In this study, prevailing poor knowledge and misconceptions of schizophrenia in the community were highlighted by healthcare officers. Studies from other countries have also reported poor to moderate levels of knowledge regarding schizophrenia in the community [37, 38]. Health seeking behavior concerning mental health problems are determined by knowledge, misconceptions, and beliefs among family members and community leaders. In developing countries, family members take patients with mental illness to traditional or religious healers owing to poor knowledge and misconceptions [39–41]. Similar findings have also been reported in developed countries like Singapore [42]. In addition, people with low literacy are more likely to seek alternate treatment for mental health problems [43, 44].

Health system related factors affected health seeking behaviors such as complicated referral system and violation of human rights within the mental health hospitals have also been reported from Indonesia [6, 44]. Violations of human rights within the health system include poor quality care and treatment, isolated and restrained patients in hospital beds, aggressive and violent behavior by hospital staff etc. [6]. Therefore, health system strengthening and enhancing community awareness should happen concurrently to avert human rights violations and delays in seeking mental healthcare services. In addition to improving accessibility, affordability, and quality of mental health services, integrating psychologists in the primary health care system is regarded as a key step towards scaling up mental health services [45, 46]. This was initiated in *Sleman* District, *Yogyakarta* Province of Indonesia in 2004 [47]. Increasing the adaptability of psychologists in the primary healthcare system and work culture differences between psychologists and healthcare providers were a few of the challenges of integrating [47].

*Pasung* in Indonesia occurs because of lack of information, access, and mental health service facilities. Of the approximately 9000 existing *Puskesmas*, only 1000 *Puskesmas* provide psychiatric services. In a total of 8 provinces in Indonesia, no mental hospital is found [48]. In addition a limited number of professionals are available, i.e., psychiatrists, mental health nurses and psychologists. There are only 600 psychiatrists and 365 clinical psychologists in Indonesia [49]. There exists a high treatment gap as indicated by high prevalence of mental disorders and inadequate mental health services. Mental health services should an integral part of primary health care. Appointing a psychologist at *Puskesmas* is one of the strategies optimize mental health services at primary healthcare level [49].

*Pasung* has been banned in Indonesia since 1977 [32]. However, it remains widespread due to enduring stigma and poor mental healthcare infrastructure and community support services [32]. Indonesia’s free from *pasung* program aims to eradicate *pasung* by 2019. Poor infrastructure, inadequate resources, and decentralized functions of the system are great hurdles to overcome in achieving this goal [32]. Political commitment and considering mental health as a top priority issue is extremely needed. Owing to wide cultural and ethical diversity in Indonesia, a socio-cultural understanding of the *pasung* practice is urgently needed. Moreover, social and cultural nuances in *pasung* practices are very strong. In addition to prohibition, concentrated efforts to educate the public are needed. Simultaneously, community engagement, especially involving religious leaders, and inter-sectoral coordination should be considered to address *pasung* and other mental health needs.

## Conclusion

*Pasung* was perceived as a necessary measure to protect patient and others from patients’ aggressive behavior. Financial constraints and dissatisfaction with existing mental health services forced family members to seek alternative or no treatment. Poor knowledge and misconceptions about schizophrenia were prevalent among family members and community leaders. Scaling up mental health services, especially in rural settings and emphasizing accessibility and quality is imperative. Health education regarding schizophrenia and prevalent misconceptions and the provision of timely and appropriate treatment is needed.

## Limitations

The small sample size may have been justified as it represented a qualitative exploratory study [50, 51]. However, the heterogeneous sample posed a limitation. This study has begun to describe the practices of society in overcoming mental disorders.

## References

[1] United Nations General Assembly: Resolution 46/119: the protection of persons with mental illness and the improvement of mental health [Internet]. http://www.un.org/documents/ga/res/46/a46r119.html. Accessed 13 Aug 2017.

[2] Schizophrenia [Internet]. World Health Organization. 2017. http://www.who.int/mediacentre/factsheets/fs397/en/. Accessed 3 July 2017.

[3] Daulima N (2014). Proses pengambilan keputusan tindakan pasung oleh keluarga terhadap klien gangguan jiwa.

[4] Memutus Rantai Tindakan Pasung di Masyarakat| UIupdate [Internet]. http://uiupdate.ui.ac.id/article/memutus-rantai-tindakan-pasung-di-masyarakat. Accessed 1 Jan 2017.

[5] Humasfik. Terapi Untuk Keluarga Terbukti Turunkan Keputusan Pasung | UIupdate [Internet]. http://uiupdate.ui.ac.id/content/terapi-untuk-keluarga-terbukti-turunkan-keputusan-pasung. Accessed 14 Aug 2017.

[6] Irmansyah I, Prasetyo YA, Minas H (2009). Human rights of persons with mental illness in Indonesia: more than legislation is needed. Int J Ment Health Syst.

[7] Wirya A (2017). Rezim Kebenaran Rasionalisme dalam Diskursus Kegilaan dan Tindakan Pendisiplinan Pasung sebagai Kejahatan. J Kriminol Indones..

[8] Firdaus F (2007). Pemenuhan Hak Atas Kesehatan Bagi Penyandang Skizofrenia di Daerah Istimewa Yogyakarta (rights fulfillment on health of people with Schizophrenia In special region of Yogyakarta). J Ilm Kebijak Huk.

[9] Andreasen N, Black D (2006). Introductory textbook of psychiatry.

[10] Canada Ms. Schizophrenia: a hand book for family. Yogyakarta: Dozz; 2005.

[11] Balitbang Kemenkes R. Riset Kesehatan Dasar; RISKESDAS. Jakarta: Balitbang Kemenkes RI; 2013.

[12] P2P. Laporan Orang dengan Gangguan Jiwa. Kabupaten Bogor: Dinas Kesehatan; 2017.

[13] Sheth H (2016). Human rights of mentally ill Clients. Int J Psychosoc Rehabil.

[14] Theodore D. Human rights in India; Mental health perspectives. 2009.

[15] Patel V (2016). Universal health coverage for schizophrenia: a global mental health priority. Schizophr Bull.

[16] Minas H, Diatri H (2008). Pasung: physical restraint and confinement of the mentally ill in the community. Int J Ment Health Syst.

[17] Puteh I, Marthoenis M, Minas H (2011). Aceh free pasung: releasing the mentally ill from physical restraint. Int J Ment Health Syst.

[18] Alem A (2000). Human rights and psychiatric care in Africa with particular reference to the Ethiopian situation. Acta Psychiatr Scand Suppl.

[19] Guan L, Liu J, Wu XM, Chen D, Wang X, Ma N (2015). Unlocking patients with mental disorders who were in restraints at home: a national follow-up study of China’s new public mental health initiatives. PLoS ONE.

[20] Tyas T. Pasung: family experience of dealing with “the deviant” in Bireuen, Nanggroe Aceh Darussalam, Indonesia. University of Amsterdam: Faculty of Social and Behavioural Sciences; 2008.

[21] Asher L, Fekadu A, Teferra S, De Silva M, Pathare S, Hanlon C (2017). “I cry every day and night, I have my son tied in chains”: physical restraint of people with schizophrenia in community settings in Ethiopia. Global Health.

[22] Read UM, Adiibokah E, Nyame S (2009). Local suffering and the global discourse of mental health and human rights: an ethnographic study of responses to mental illness in rural Ghana. Global Health.

[23] “Like a death sentence”: abuses against persons with mental disabilities in Ghana. In Human rights watch; 2012.

[24] Marrow J, Luhrmann T (2012). The zone of social abandonment in cultural geography: on the street in the United States, inside the family in India. Cult Med Psychiatry.

[25] Ekawati FM, Claramita M, Hort K, Furler J, Licqurish S, Gunn J (2017). Patients’ experience of using primary care services in the context of Indonesian universal health coverage reforms. Asia Pac Fam Med.

[26] Ramacciotti CE, Paoli RA, Catena M, Ciapparelli A, Dell’Osso L, Schulte F (2004). Schizophrenia and binge-eating disorders. J Clin Psychiatry.

[27] Lundgren JD, Allison KC, Crow S, O’Reardon JP, Berg KC, Galbraith J (2006). Prevalence of the night eating syndrome in a psychiatric population. Am J Psychiatry.

[28] Lundgren JD, Rempfer MV, Brown CE, Goetz J, Hamera E (2010). The prevalence of night eating syndrome and binge eating disorder among overweight and obese individuals with serious mental illness. Psychiatry Res.

[29] Palmese LB, DeGeorge PC, Ratliff JC, Srihari VH, Wexler BE, Krystal AD (2011). Insomnia is frequent in schizophrenia and associated with night eating and obesity. Schizophr Res.

[30] Palmese LB, Ratliff JC, Reutenauer EL, Tonizzo KM, Grilo CM, Tek C (2013). Prevalence of night eating in obese individuals with schizophrenia and schizoaffective disorder. Compr Psychiatry.

[31] Kouidrat Y, Amad A, Lalau J-D, Loas G (2014). Eating Disorders in Schizophrenia: implications for research and management. Schizophr Res Treat.

[32] Home Movement for Global Mental Health [Internet]. http://www.globalmentalhealth.org/. Accessed 15 Sep 2017.

[33] Joint Commission on Accreditation of Health Care Organization: Restraint and Seclusion Standards. Comprehensive Accreditation Manual for Hospitals. Oakbrook Terrace, Illinois: JCAHO; 2001.

[34] Drew N, Funk M, Tang S, Lamichhane J, Chávez E, Katontoka S (2011). Human rights violations of people with mental and psychosocial disabilities: an unresolved global crisis. Lancet.

[35] Maj M (2011). Report on the implementation of the WPA action plan 2008–2011. World Psychiatry.

[36] Chisholm D, Flisher AJ, Lund C, Patel V, Saxena S, Lancet Global Mental Health Group (2007). Scale up services for mental disorders: a call for action. Lancet.

[37] Wong FKD, Lam YKA, Poon A (2010). Knowledge and preferences regarding schizophrenia among Chinese-speaking Australians in Melbourne, Australia. Soc Psychiatry Psychiatr Epidemiol.

[38] Economou M, Richardson C, Gramandani C, Stalikas A, Stefanis C (2009). Knowledge about schizophrenia and attitudes towards people with schizophrenia in Greece. Int J Soc Psychiatry.

[39] Coton X, Poly S, Hoyois P, Sophal C, Dubois V (2008). The healthcare-seeking behaviour of schizophrenic patients in Cambodia. Int J Soc Psychiatry.

[40] Jacob K (2001). Community care for people with mental disorders in developing countries: problems and possible solutions. Br J Psychiatry.

[41] Girma E, Tesfaye M (2011). Patterns of treatment seeking behavior for mental illnesses in Southwest Ethiopia: a hospital based study. BMC Psychiatry.

[42] Chong S, Mythily, Lum A, Chan Y, McGorry P (2005). Determinants of duration of untreated psychosis and the pathway to care in Singapore. Int J Soc Psychiatry.

[43] Jorm A (2012). Mental health literacy: empowering the community to take action for better mental health. Am Psychol.

[44] Marthoenis M, Aichberger M, Schouler-Ocak M (2016). Patterns and determinants of treatment seeking among previously untreated psychotic patients in Aceh province, Indonesia: a qualitative study. Scientifica.

[45] Hass LJ (2004). Handbook of primary care psychology.

[46] Setiyawati D, Colucci E, Blashki G, Wraith R, Minas H (2014). International experts’ perspectives on a curriculum for psychologists working in primary health care: implication for Indonesia. Health Psychol Behav Med.

[47] Retnowati S. Psikolog di Puskesmas, Solusi Kesehatan Jiwa di Indonesia [Internet]. Universitas Gadjah Mada. 2001. https://ugm.ac.id/id/berita/3646-psikolog.di.puskesmas.solusi.kesehatan.jiwa.di.indonesia?fb_comment_id=569327626451003_881343935249369#f96237840d884. Accessed 28 Mar 2018.

[48] Maharani D. Pemasungan, Cermin Buruknya Pelayanan Kesehatan Jiwa. 2014.

[49] Agung. Psikolog di Puskesmas, Solusi Kesehatan Jiwa di Indonesia. 2011.

[50] Crouch M, McKenzie H (2006). The logic of small samples in interview-based qualitative research. Soc Sci Inf.

[51] Guest G, Bunce A, Johnson L (2006). How many interviews are enough?. Field Methods.

